# Association of postexercise heart rate recovery with body composition in healthy male adults: Findings from Pakistan

**DOI:** 10.1111/anec.12711

**Published:** 2019-10-08

**Authors:** Fahad Azam, Abida Shaheen, Khurram Irshad, Afrose Liaquat, Hania Naveed, Saeed Ullah Shah

**Affiliations:** ^1^ Pharmacology & Therapeutics Shifa College of Medicine Shifa Tameer‐e‐Millat University Islamabad Pakistan; ^2^ Department of Basic Health Sciences, Pharmacology Section Shifa College of Medicine Islamabad Pakistan; ^3^ Physiology Shifa College of Medicine Shifa Tameer‐e‐Millat University Islamabad Pakistan; ^4^ Department of Basic Health Sciences, Physiology Section Shifa College of Medicine Islamabad Pakistan; ^5^ Biochemistry Shifa College of Medicine Shifa Tameer‐e‐Millat University Islamabad Pakistan; ^6^ Department of Basic Health Sciences, Biochemistry Section Shifa College of Medicine Islamabad Pakistan; ^7^ Pathology Shifa College of Medicine Shifa Tameer‐e‐Millat University Islamabad Pakistan; ^8^ Department of Basic Health Sciences, Pathology Section Shifa College of Medicine Islamabad Pakistan; ^9^ Consultant Cardiologist Shifa International Hospital Islamabad Pakistan; ^10^ Department of Cardiology Shifa International Hospital Islamabad Pakistan

**Keywords:** BMI, body fat ratio, heart rate recovery, exercise, postexercise heart rate, metabolic risk factors

## Abstract

**Background:**

This study investigated the effect of body mass index (BMI) and body fat ratio with postexercise heart rate recovery (HRR) after 2 minutes of rest.

**Methods:**

Sixty‐four healthy males aged between 25 and 55 years participated in the study. BMI, body fat ratio, waist circumference, and physical activity were recorded. Peak heart rate after exercise and HRR after 2 min of rest were obtained.

**Results:**

Mean age of participants was 35.53 ± 6.57. Mean BMI and HRR were 25.06 ± 4.62 and 26.07 ± 7.43, respectively. BMI and body fat ratio had significant negative correlation with HRR with *r* values of −.833 and −.877, respectively (*p* < .001*). Linear regression showed BMI and body fat ratio with significant coefficient of −0.426 (*p* = .04*) and −0.627 (*p* < .001*) with HRR, respectively. Participants with BMI ˂ 25 had higher HRR in comparison to participants with BMI ≥ 25 (*p* < .001*). Participants with body fat ratio of ˂25 had significantly higher HRR of 35.9 ± 3.98 in comparison to participants with body fat ratio ≥ 25 (*p* = <.001*).

**Conclusion:**

Body mass index and body fat ratio are strong predictors of HRR in Pakistani healthy male adults, suggesting a strong link between metabolic risk factors and impaired autonomic nervous system.

## 1 INTRODUCTION

Postexercise heart rate (PEHR) is an established measure of autonomic nervous system function and is used as a prognostic tool to assess and predict cardiovascular morbidity and mortality. Variation in heart rate (HR) during exercise is believed to be due to the activation of sympathetic autonomic nervous system (SANS) and disinhibition of parasympathetic autonomic nervous system (PANS). The quick drop in heart rate immediately after cessation of physical activity results mainly due to the increase in cardiac vagal activity, and the subsequent slow recovery of the heart rate results from vagal inhibition as well as slowly decreasing SANS activity (Dimkpa, 2009; Ogoh et al., 2005). Generally, recovery of heart rate equal to or more than 12–22 beats after first minute of termination of exercise is considered normal whereas heart rate recovery after 2 min of rest is considered a strong predictor of cardiovascular system associated morbidity and mortality (Cole, Blackstone, Pashkow, Snader, & Lauer, 1999; Lipinski, Vetrovec, & Froelicher, 2004; Sydó et al., 2018). Overall, a very rapid HR recovery immediately after exercise is associated with lower risk of cardiovascular diseases, independent of other exercise parameters (Meibodi, Larson, Levy, O'Donnell, & Vasan, 2002).

A number of studies have highlighted correlation of slow heart rate recovery (HRR) with obesity, socioeconomic status, depression and increased risk of hypertension, diabetes, and metabolic syndrome (Kanjilal et al., 2006; Qiu et al., 2017). Moreover, considering the global scourge of metabolic syndrome in recent years characterized by insulin resistance, abdominal obesity, hypertension, and hyperlipidemia, there is need of proactive approaches to identify population at risk (Hanifah et al., 2013; Saklayen, 2018).

Body mass index (BMI) has traditionally been used as a popular screening tool for obesity, and values of 20–24.9 have been considered normal. However, it is becoming increasingly evident that BMI is an over simplistic tool to screen for obesity and recent tools such as body fat ratio have better prognostic and predictive value to assess degree of obesity (Liu, Ma, Lou, & Liu, 2013; Nuttall, 2015). There is controversy over acceptable normal reference ranges for body fat ratio of males and females in different age groups; however, there is general consensus that body fat ratio <25% of body mass is considered normal in males up to 50 years of age (Ho‐Pham, Campbell, & Nguyen, 2011).

With this background, the present study aims to investigate the association of HRR with body composition measurements, BMI, smoking, and physical activity and to ascertain potential contributors responsible for slow HRR in Pakistani healthy adult population.

## 2 METHODS

A cross‐sectional study was conducted on sixty‐four healthy adult males at Shifa College of Medicine, Shifa Tameer‐e‐Millat University Islamabad, Pakistan. Ethical approval was obtained from institutional review board (IRB), and study was conducted in accordance with guidelines of International Conference on Harmonization for Good Clinical Practice (ICH‐GCP) and the Declaration of Helsinki.

Healthy male volunteers aged between 25 and 55 years and working as staff members at medical college campus were included in the study. Volunteers with any ischemic or congenital cardiovascular disease, musculoskeletal injuries, respiratory insufficiency, or any other chronic illness were excluded from the study.

Participants were informed about the nature of the study, and written informed consent was taken from study participants. All participants were asked to fast overnight and to avoid strenuous physical activity 1 day prior to data collection. Participants were asked to empty their bladders 30 min before recording of observations. Height in cm was measured by a calibrated vertical stadiometer. During height measurement, participants were to remove their shoes and socks. Body mass index (BMI) was calculated by using weight in kilogram divided by height in meters squared. Waist circumference was measured in inches rounded off to the nearest decimal.

Body fat of each participant was measured by using a leg‐to‐leg bioelectrical impedance commercial glass body composition analyzer (Beurer living glass diagnostic scale BG‐42). Subjects were asked to stand vertically with bare feet on contact footpad electrodes, and body composition such as fat mass, lean mass, and water percentage was recorded simultaneously by preprogrammed calculations automatically done by analyzer.

Pulse rate, systolic blood pressure (SBP), and diastolic blood pressure (DBP) of all participants were recorded in a fully relaxed state before start of exercise by trained medical persons. Baseline pulse rate at resting state was recorded both manually and verified by a wearable fitness tracker (Fitbit Charge 3 device) which utilizes the principle of light‐emitting diodes. Participants were asked to wear the fitness tracker throughout the experiment. Exercise test was performed in the presence of trained physiologist. Participants were asked to perform a standardized exercise by walking for one hundred and fifty steps with ten‐kilogram heavy dumbbells in each hand. Participants were asked to stop exercise immediately in case of feeling discomfort or breathlessness. Pulse rate for each participant was immediately recorded after completion of exercise and then after a rest of 2 min manually and by using a wearable fitness tracker. Heart rate recovery (HRR) for each individual was obtained by computing the difference between pulse rate immediately after exercise and pulse rate after 2 min of rest in sitting position.

### 2.1 Statistical analysis

Obtained data were entered in MS Excel software and analyzed by IBM's Statistical Package for the Social Sciences (SPSS) version 23 (IBM). Participants’ baseline characteristics and exercise parameters were computed as mean and standard deviation, whereas frequencies and percentages were used for qualitative variables. Correlation between HRR and body composition measurements was computed by Pearson's correlation analysis, and multiple regression analysis was utilized to find out standardized coefficients after controlling for age, resting heart rate, and blood pressure before exercise. Independent *“t”* test was used to analyze difference in HRR among participants with different BMI, body fat ratio, number of hours of sleep, and smoking pattern. ANOVA was used to analyze differences among participants with different patterns of physical activity. *p* value <.05 was considered to be significant.

## 3 RESULTS

Sixty‐four male healthy volunteers participated in the study. Baseline characteristics, body measurements, and pre‐exercise parameters are given in Table 1. Twelve (18.8%) out of 64 participants were smokers.

**Table 1:** Baseline characteristics and pre‐exercise parameters of participants (*n* = 64)

Baseline characteristics	Mean ± *SD*
Age (years)	35.53 ± 6.57
SBP before exercise (mm Hg)	122.59 ± 14.24
DBP before exercise (mm Hg)	84.78 ± 10.39
Pulse rate before exercise (beats/min)	74.53 ± 9.68
Height (cm)	171.53 ± 5.27
Weight (kg)	74.53 ± 11.47
BMI (kg/m^2^)	25.06 ± 4.62
Body fat %	26.07 ± 7.43
Waist circumference (cm)	94.06 ± 12.48
Peak heart rate after exercise (beats/min)	98.93 ± 9.99
PEHR after 2‐min rest (beats/min)	70.43 ± 11.25
Average recovery after 2‐min rest (beats/min)	28.5 ± 7.24
Average sleep duration (hr)	7.23 ± 0.77

Note

Data expressed as mean ± *SD*.

Abbreviations: BMI, body mass index; DBP, diastolic blood pressure; HRR, heart rate recovery; SBP, systolic blood pressure.

Table 2 shows correlation between the slow HRR and potential indicators. BMI, body fat ratio, and waist circumference were inversely correlated and showed significant correlation with HRR. Body fat ratio and BMI had the strongest association with HRR. Multiple regression analysis showed body fat and BMI inverse correlation with HRR (*p* = .000 and *p* = .04, respectively). All data were adjusted for age, resting pulse rate, and blood pressure (Table 2).

**Table 2:** Pearson's correlation analysis of HRR with age, body composition parameters, and sleep and linear regression analysis between HRR with body composition parameters and sleep duration after controlling for age, resting heart rate, and blood pressure

Variables	Pearson's correlation		Linear regression analysis	
	*r*	*p*‐value	β	*p*‐value
Age	−.355	.002*		
BMI	−.833	<.001*	−0.426	.04*
Body fat %	−.877	<.001*	−0.627	<.001*
Waist circumference	−.758	<.001*	−0.02	.717
Sleep duration	.267	.016*	0.91	.19

* = *p*‐value less than 0.05.

Significant differences in 2‐min HRR were observed in males with BMI ≥ 25, body fat ratio ≥ 25, and different patterns of physical activity. These results are shown in Table 3.

**Table 3:** Mean HRR levels in participants with different status of physical activity, BMI, body fat ratio, sleep, and smoking (*n* = 64)

Variables	No. of patients (%)	HRR after 2‐min exercise (Mean ± *SD*)	*p*‐value
BMI < 25	30 (46.8)	33.46 ± 6.13	<.001*
BMI ≥ 25	34 (53.1)	24.11 ± 4.99	
BMI < 23	22 (34.37)	34.00 ± 6.56	<.001*
BMI ≥ 23	42 (25.61)	25.61 ± 5.82	
Body fat ratio < 25	22 (34.37)	35.9 ± 3.98	<.001*
Body fat ratio ≥ 25	42 (25.61)	24.61 ± 5.25	
Sleep ≥ 8 hr	28 (43.75)	30.21 ± 8.94	.09
Sleep < 8 hr	36 (56.25)	27.16 ± 5.34	
Nonsmokers	52 (81.25)	28.80 ± 7.63	.093
Smokers	12 (18.75)	27.16 ± 5.30	
Regular physical activity	10 (15.62)	36.20 ± 6.37	<.001*
Occasional physical activity	18 (28.12)	28.77 ± 5.44	
Sedentary life style	36 (56.25)	26.22 ± 6.88	

* = *p*‐value less than 0.05.

## 4 DISCUSSION

Our present study investigated the association between body composition measurements and other potential indicators with 2 min postexercise heart rate (PEHR) in sixty‐four healthy male adult population. Participants with increased body fat ratio, higher BMI, and sedentary lifestyle showed a strong correlation with decreased 2‐min HRR.

Out of sixty‐four males, forty‐two (56.2%) participants had inverse correlation between body fat percentage and HRR after 2‐min exercise (<0.001). Moreover, 53.1% participants with BMI ≥ 25 showed statistically significant slow HRR after exercise test. Using multiple linear regression analysis, all major variables showed negative standardized coefficient in comparison with HRR but the most significant impact was again observed with body fat ratio and BMI with highly significant *p* values. Studies conducted by different researchers have also reported heart rate variability in overweight adults and confirm our findings that variations in the body compositions can reduce the adaptive flexibility of autonomic cardiac function (Hanifah et al., 2013; Qiu et al., 2017; Triggiani et al., 2017). Low physical activity has also been documented as an independent variable of diminished heart rate recovery in obese adults. HRR deteriorations are cholinergic and are associated with obesity‐induced immune response, unstable glucose regulation, inflammatory adipokines secretion, and low physical activity which ultimately leads to adverse cardiac outcomes and increased risk of morbidity and mortality (Lampert et al., 2008; Liao et al., 2017; Lopes & Egan, 2006).

Altered heart rate recovery has been investigated in various studies with diverse results. In our study, significant changes in HRR with increased waist circumference were observed and it was found to be inversely related to HRR after 2 min. Results of our study are in accordance with another study that proved the waist circumference as the only parameter associated with impaired PEHR parameters (Lin et al., 2008).

Physical activity has an established association with HRR due to augmented tone of parasympathetic autonomic nervous system. Our findings showed strong association of decreased heart rate recovery in individuals with sedentary lifestyle as compared to the ones with occasional or regular physical activity. A study conducted on middle‐aged adults revealed that persons with habitual moderate physical activity had a better overall health status as compared to persons with low physical activity (Buchheit et al., 2005).

The number of smokers in our study was quite less compared with the number of nonsmokers. Nonsmokers in our study had higher PEHR in comparison with smokers but this difference was statistically not significant. The probable reason for this observation could be the fact that this study was conducted in a medical college campus and the volunteers being staff of a medical institution probably had better awareness about hazards of smoking as compared to the general population. Similarly, high awareness of these participants about healthy lifestyle is also evident from the fact that their mean body fat and average number of hours of daily sleep were quite close to the international recommended guidelines. Our results are in contrast to the results of a study conducted to see the effects of exercise on heart rate recovery of smokers after exercise in which the heart rate recovery of smokers was significantly higher in comparison with the nonsmokers (Papathanasiou et al., 2013).

Limitations of the study include noninclusion of females in whom potential indicators for heart recovery could be different due to unsimilar body fat distribution and hormonal influence on heart rate variability indices. Similar studies are required to investigate major risk factors in female adults of Pakistani population.

## 5 CONCLUSION

Body mass index, body fat ratio, and waist circumference showed inverse correlation with 2‐min HRR in healthy Pakistani adult males. Body fat ratio and BMI were strong predictors of heart rate recovery in this population. Preventive strategies need to be identified which can help in early detection of disease risk, and heart rate recovery has the potential to serve as a widely used tool for early risk detection.

## CONFLICTS OF INTEREST

The author(s) declare no potential conflicts of interest with respect to the study, authorship, and/or publication of this article.

## AUTHOR CONTRIBUTIONS

FA and AS conceived, designed, collected data, did statistical analysis, and drafted manuscript. AS and KIQ drafted manuscript, reviewed, and did final approval of manuscript. AL, HN and SUS conceived and designed study, collected data, and reviewed manuscript.
